# Rapamycin-induced hyperglycemia is associated with exacerbated age-related osteoarthritis

**DOI:** 10.1186/s13075-021-02637-1

**Published:** 2021-10-07

**Authors:** Dennis M. Minton, Christian J. Elliehausen, Martin A. Javors, Kelly S. Santangelo, Adam R. Konopka

**Affiliations:** 1 Division of Geriatrics and Gerontology, Department of Medicine, University of Wisconsin-Madison, Madison, Wisconsin USA; 2 Department of Kinesiology, University of Illinois at Urbana-Champaign, Champaign, Illinois USA; 3 Departments of Psychiatry and Pharmacology, University of Texas Health Science Center at San Antonio, San Antonio, Texas USA; 4 Department of Microbiology, Immunology, Pathology, Colorado State University, Fort Collins, Colorado USA; 5 Geriatric Research, Education, and Clinical Center, William S. Middleton Memorial Veterans Hospital, Madison, Wisconsin USA

**Keywords:** Aging, mTOR, AMPK, Dunkin Hartley guinea pig, Primary osteoarthritis

## Abstract

**Background:**

The objective of this study was to determine if mechanistic target of rapamycin (mTOR) inhibition with or without AMP-activated protein kinase (AMPK) activation can protect against primary, age-related OA.

**Design:**

Dunkin-Hartley guinea pigs develop mild primary OA pathology by 5 months of age that progresses to moderate OA by 8 months of age. At 5 months, guinea pigs served as young control (*n* = 3) or were fed either a control diet (*n* = 8), a diet enriched with the mTOR-inhibitor rapamycin (Rap, 14 ppm, *n* = 8), or Rap with the AMPK-activator metformin (Rap+Met, 1000 ppm, *n* = 8) for 12 weeks. Knee joints were evaluated by OARSI scoring, micro-computed tomography, and immunohistochemistry. Glenohumeral articular cartilage was collected for western blotting.

**Results:**

Rap- and Rap+Met-treated guinea pigs displayed lower body weight than control. Rap and Rap+Met inhibited articular cartilage mTORC1 but not mTORC2 signaling. Rap+Met, but not Rap alone, stimulated AMPK. Despite lower body weight and articular cartilage mTORC1 inhibition, Rap- and Rap+Met-treated guinea pigs had greater OA severity in the medial tibial plateau due to articular cartilage structural damage and/or proteoglycan loss. Rap and Rap+Met increased plasma glucose compared to control. Plasma glucose concentration was positively correlated with proteoglycan loss, suggesting hyperglycemic stress after Rap treatment was related to worsened OA.

**Conclusions:**

This is the first study to show that Rap induced increase in plasma glucose was associated with greater OA severity. Further, articular cartilage mTORC1 inhibition and bodyweight reduction by dietary Rap and Rap+Met did not appear to protect against primary OA during the prevailing hyperglycemia.

**Supplementary Information:**

The online version contains supplementary material available at 10.1186/s13075-021-02637-1.

## Background

Primary, age-related osteoarthritis (OA) is estimated to account for as many as 90% of all knee OA cases in humans [1]. However, preclinical research commonly relies on experimental models of secondary OA. Although primary and secondary OA share similar pathological outcomes, there is a growing body of evidence to suggest they are driven by distinct mechanisms. Retrospective analysis of differentially expressed genes from separate cohorts of primary and secondary OA patients relative to their healthy controls found that only 10% of differentially upregulated and 35% of differentially downregulated genes in OA vs non-OA samples are conserved between primary and secondary OA [2, 3]. Therefore, 65–90% of differentially expressed genes may be unique to primary versus secondary OA. Additionally, transgenic animal models have revealed that several genes are differentially involved in the progression of primary and secondary OA [4–9]. For example, deletion of *Panx3* protects against surgically induced OA yet dramatically worsens primary OA [4] and deletion of *JNK1/2* accelerates the development of primary OA while having no effect on surgically induced OA progression [9]. Together, these studies reinforce that unique mechanisms underpin these two forms of OA.

Age is one of the greatest risk factors for nearly every chronic disease, including primary OA. Two evolutionarily conserved kinases, mechanistic target of rapamycin (mTOR) and AMP-activated protein kinase (AMPK), are energy sensing pathways similarly dysregulated during aging and OA [10–13]. Providing the mTOR inhibitor rapamycin (Rap) in the diet can extend lifespan in mice and delay the onset of several age-related morbidities [12, 14]. However, despite slowing aging and delaying the onset of select age-related pathologies, Rap also has several notable side effects, including hyperglycemia, new onset diabetes, and dyslipidemia [15]. The anti-diabetic drug metformin (Met) can activate AMPK and, when added to Rap, extends lifespan to a greater extent than historical cohorts of mice treated with Met or Rap alone and helps mitigate Rap-induced glucose intolerance [16, 17]. While the prospect of lifespan extension is tantalizing, extending lifespan without delaying the onset or slowing the progression of the most debilitating age-associated conditions could be viewed as detrimental. Therefore, it is imperative to understand if purported lifespan-extending therapies that target the fundamental biology of aging are also capable of delaying the onset of chronic diseases, such as primary OA.

mTOR exists as complex I (mTORC1) and complex II (mTORC2). mTORC1 regulates cellular proliferation, protein synthesis, senescence, and survival while mTORC2 functions downstream of insulin signaling on substrates such as Akt [12]. In articular cartilage, mTORC1 activity increases with age and is sufficient to induce OA in young male mice [10]. In non-articular tissues, acute or intermittent Rap selectively inhibits mTORC1 while chronic Rap administration also inhibits mTORC2 activity [18]. Cartilage-specific deletion of the mTOR kinase and systemic or intra-articular injections of Rap and the mTORC1/2 inhibitor Torin 1 lower surgically induced OA in young-male mice and rabbits [19–22]. While these findings support mTOR-based therapeutics for OA, the completed studies were exclusively in injury-induced models of OA and have not been investigated in primary, age-related OA.

Recently, it has been proposed that the positive effects of mTOR inhibition on OA pathology may be diminished by feedback activation of PI3K and has raised questions about the need for a dual treatment strategy that inhibits both mTOR and upstream PI3K signaling [23, 24]. In addition to activating AMPK, Met has pleotropic effects including inhibition of PI3K signaling in rheumatoid arthritis fibroblast-like synoviocytes [25]. Moreover, Met and other AMPK-activators have chondroprotective effects against inflammatory-induced protease expression in vitro [26, 27] and protect against injury-induced OA in young male mice and rhesus monkeys [28]. Treatment with Met is also is associated with a lower rate of medial tibiofemoral cartilage volume loss and risk of total knee replacement in obese patients [29]. However, Met as an adjuvant therapy to Rap has not been investigated in primary OA.

The Dunkin-Hartley guinea pig is a well-characterized outbred model of primary OA. The progression of OA in guinea pigs is related to bodyweight [30] and shares a similar age-related and spatial progression to humans [31]. Additionally, changes in gene and protein expression in guinea pigs largely mimic those seen during OA progression in humans [32–37]. Mild OA pathology develops by 5 months in guinea pigs that progresses to moderate OA by 8–9 months of age [31, 38, 39]. Therefore, at 5 months of age, we treated guinea pigs with lifespan-extending doses of dietary Rap (14 ppm) or a combination of dietary Rap+Met (14 + 1000 ppm) for 12 weeks to slow the progression from mild to moderate OA. This study is the first to evaluate if lifespan extending treatments can modify primary OA, the most prevalent form of OA observed in older adults.

## Methods

### Animal use

All tissues were collected at the University of Illinois Urbana-Champaign and approved by the Institutional Animal Care and Use Committee. Data collection and analysis were completed at University of Wisconsin-Madison and William S. Middleton Memorial Veterans Hospital. Because male Dunkin-Hartley guinea pigs develop more severe OA pathology than female [40], we used male animals to maximize the potential for the interventions to slow the progression of OA. Similar to previous work [41], male Dunkin-Hartley guinea pigs (Charles River) were singly housed in clear plastic, flat bottomed cages (Thoren, model #6) with bedding. Guinea pigs were single housed to measure food consumption. Twelve-hour light/dark cycles were used beginning at 0600. Guinea pigs acclimated for 2–3 weeks and were provided standard chow diet (Evigo 2040) fortified with vitamin C (1050 ppm) and vitamin D (1.5 IU/kg) and water ad libitum until 5 months of age. Guinea pigs were then euthanized to serve as young control (*n* = 3) or randomized to continue the standard diet without eudagrit microcapsules (*n* = 8), or receive standard diets enriched with eudagrit encapsulated rapamycin (14 ppm, *n* = 8) or the combination of encapsulated rapamycin plus metformin (14 + 1000 ppm, *n* = 8) for 12 weeks. Guinea pigs were randomized to match bodyweight between groups prior to beginning treatment. Diets were enriched with microencapsulated rapamycin (Rapamycin holdings) and/or metformin (AK Scientific, I506) at concentrations previously shown to extend lifespan in mice [14, 16, 42]. Food consumption was recorded on Monday, Wednesday, and Friday between 8 and 9 AM, and body weight was recorded before feeding on Monday. Guinea pigs treated with Rap (14 ppm) or Rap+Met (14 + 1000 ppm) diet had ad libitum access to food. Dietary Rap (14 ppm) treatment has been shown to significantly reduce bodyweight in mice [17, 43]. Therefore, we matched food consumption in the control group to the Rap diets to minimize the influence of food intake on dependent variables. One guinea pig in the Rap+Met group was euthanized early due to a wound on the gums which led to suppressed appetite and infection. Tissues from this animal were not collected for analysis. It could not be determined if this was due to a laceration or an oral ulcer, the latter of which is a known side effect of mTOR inhibitors [44].

### Tissue collection

Two animals were sacrificed daily between 7 and 10 AM. Food and water were removed from the cages 2–4 h before euthanasia. Animals were anesthetized in a chamber containing 5% isoflurane gas in oxygen and maintained using a face mask with 1.5–3% isoflurane. Blood was collected by cardiac puncture followed by excision of the heart. The right hind limb was removed at the coxofemoral joint, fixed in 10% neutral buffered formalin (NBF) for 48 h, and transferred to 70% ethanol until processed for histology. Glenohumeral cartilage was collected, snap frozen in liquid nitrogen, and stored at – 80 °C for further analysis. Because testicular atrophy has been observed following Rap treatment [45], the left testicle was preserved in 10% NBF and weighed. Although tissues are commonly weighed before fixation, previous work demonstrates that fixation negligibly effects testicle weight in similarly sized animals [46].

### Analysis of experimental diets and blood

Twenty-five-milligram samples of diets enriched with Rap (14 ppm) or the combination of Rap+Met (14 + 1000 ppm), and aliquots of whole blood (*n* = 4 per group) were sent to the Bioanalytical Pharmacology Core at the San Antonio Nathan Shock Center to confirm drug concentrations in the diet and in circulation. Analysis was performed using tandem HPLC-MS as described previously [14, 47, 48]. Frozen aliquots of plasma were thawed to measure glucose and lactate concentrations using the YSI Biochemistry Analyzer (YSI 2900).

### Micro-computed tomography (μCT)

Right hind limbs from half of each treatment group (*n* = 4 per group) were scanned using a Rigaku CT Lab GX130 at 120 μA and 110 kV for 14 min, achieving a pixel size of 49 μm. Scans were first processed in Amira 6.7 (ThermoFisher) where epicondylar width was measured and a series of dilation, erosion, filling, and image subtraction functions were used to isolate trabecular and cortical bone as described previously [49]. Scans were then resliced 4 times along axes perpendicular to medial and lateral tibial and femoral articular surfaces and binarized using identical thresholds. NIH ImageJ software and BoneJ plugin were used to quantify thickness, spacing, and volume fraction measurements. Cortical thickness was measured by placing polygonal regions of interest (ROI) in resliced scans to encompass the articular surfaces in each joint compartment. Trabecular thickness, spacing, and bone volume fraction were measured by placing transverse ROIs (2.4 × 2.4 × 1 mm) in the trabecular bone of each joint compartment.

### Histology

Knee joints were decalcified in a 5% ethylenediaminetetraacetic acid, changed every 2–3 days for 6 weeks. Joints were then cut in a coronal plane along the medial collateral ligament, paraffin embedded, and sectioned at 5 μm increments for Toluidine Blue staining and immunohistochemistry (IHC). Slides were scanned using the Hamamatsu NanoZoomer Digital Pathology System, providing 460 nm resolution. Scan focus points were set manually along the articular cartilage. Imaged slides were then scored by two blinded reviewers for OA severity following OARSI modified Mankin guidelines as described [39]. Briefly, toluidine blue stained histology slides were assigned scores for severity of articular cartilage structural damage (0–8), proteoglycan content loss as assessed by absence of toluidine blue staining (0–6), disruption of chondrocyte cellularity (0–3), and tidemark integrity (0–1), with a total possible score of 18 per joint compartment (Total OARSI Score). One guinea pig each from the Rap and Rap+Met groups was unable to be analyzed due to off-axis transection before embedding. One control animal was a statistical outlier as detected by Grubb’s test and was excluded from the study. Therefore, *n* = 7 per group were used for histopathological analysis.

### Immunohistochemistry

Antigen retrieval was performed in 10 mM sodium citrate for 7 h at 60 °C. Endogenous peroxidase activity was quenched using 3% H_2_O_2_ for 15 min before blocking in 5% normal goat serum diluted in TBST for 1 h at RT. Slides were incubated overnight in 200–300 μL of either p-RPS6 (1:200 dilution; Cell Signaling, 4858) or a rabbit IgG isotype control (Cell Signaling, 3900) diluted to match primary antibody concentration. Primary antibodies against p-Akt Ser473 (1:100 dilution; 4060) and p-AMPK Thr172 (1:200 dilution; 50081) from Cell Signaling were attempted, but reactivity was not seen in guinea pig articular cartilage. One hundred and fifty to 200 μL of goat anti-rabbit secondary antibody (Cell Signaling, 8114) was added for 1 h at room temperature followed by exposure in 3,3′-diaminobenzadine (DAB; Cell Signaling, 8059) for 10 min. Slides were then counterstained using hematoxylin, dehydrated, and cleared through graded ethanol and xylene, coverslipped using Permount (Electron Microscopy Sciences), and viewed and imaged under a brightfield microscope. No DAB staining was seen following incubation with the IgG control or secondary antibody alone, confirming specificity of the primary antibody. For quantification, ROIs were placed to encompass areas of staining in the medial tibial articular cartilage, and cells were counted to determine the percent-positive cells. For intensity-based quantification, a color deconvolution for DAB staining was applied in ImageJ, and mean integrated intensity was quantified by averaging two p-RPS6 replicates and subtracting background staining of IgG controls.

### Western blot

Cartilage was removed from the glenohumeral joint using a scalpel and placed in reinforced Eppendorf tubes containing 500 mg of ceramic beads (Fisher, 15-340-160) and 200 μL of RIPA buffer with protease and phosphatase inhibitors (Sigma, 5892970001), and homogenized by 2, 30-s cycles at 6 m/s in the Omni BeadRuptor. Homogenate was transferred to microcentrifuge tubes and spun at 10,000×*g* for 10 min at 4 °C. Supernatants were diluted to equal concentration following a BCA assay. Samples were prepared in reducing conditions with β-mercaptoethanol in 4x Laemmli Sample Buffer (BioRad, 1610747) and heated at 95 °C for 5 min. Ten micrograms of protein was separated on 4–15% TGX precast gels (BioRad, 4561083) and transferred to PVDF membranes (BioRad, 1620177). Membranes were blocked in TBST with 5% bovine serum albumin (Sigma, A9647) for 1 h at RT and incubated overnight at 4 °C in primary antibodies against p-RPS6 Ser235/236 (4858), RPS6 (2217) p-Akt Ser473 (4060), Akt (4685), P-AMPK Thr172 (50081), AMPK (2532), and LC3B (3868) from Cell Signaling and ADAMTS5 (ab41037), MMP13 (ab39012), and b-Actin (ab8226) from Abcam. HRP-conjugated anti-Rabbit (Cell Signaling) or anti-Mouse (Abcam) secondary antibodies were diluted 1:5000 for all proteins except b-Actin (1:10,000 dilution). All membranes were imaged using a UVP BioSpectrum 500 (UVP) following 5-min incubation in a 2:1 combination of SuperSignal Pico (Fisher, 34577) and Femto (Fisher, PI34095) chemiluminescent substrates except b-Actin which received Pico alone. Densitometric analysis was performed using VisionWorks (Analytikjena). Phosphorylated proteins are expressed relative to their total protein and other targets are expressed relative to b-Actin.

### Statistical analysis

Previous work demonstrated that a sample size of *n* = 6 is adequately powered to detect changes between groups in guinea pigs [41]. Therefore, we a priori determined our sample size (*n* = 7–8 per group) to be appropriate to detect differences between treatment groups. All data were subjected to normality testing via the Shapiro-Wilk test. A two-way repeated measures ANOVA (time × treatment) was performed to determine differences in food consumption and body weight. For all other variables, a one-way ANOVA comparing the difference between each treatment and 8-month controls was performed. Upon a significant interaction, Holm-Sidak’s multiple comparisons test was used. Data with non-Gaussian distribution were compared using non-parametric Mann-Whitney tests or the Kruskal-Wallis test followed by Dunn’s multiple comparisons test and noted in the figure legend. Pearson’s R was used to determine correlation between variables. *P*-values < 0.05 were considered statistically significant. Data are presented as scatter plots with mean or mean ± standard deviation (SD).

## Results

### Influence of rapamycin and rapamycin+metformin on guinea pig physical and metabolic characteristics

**Fig. 1:**
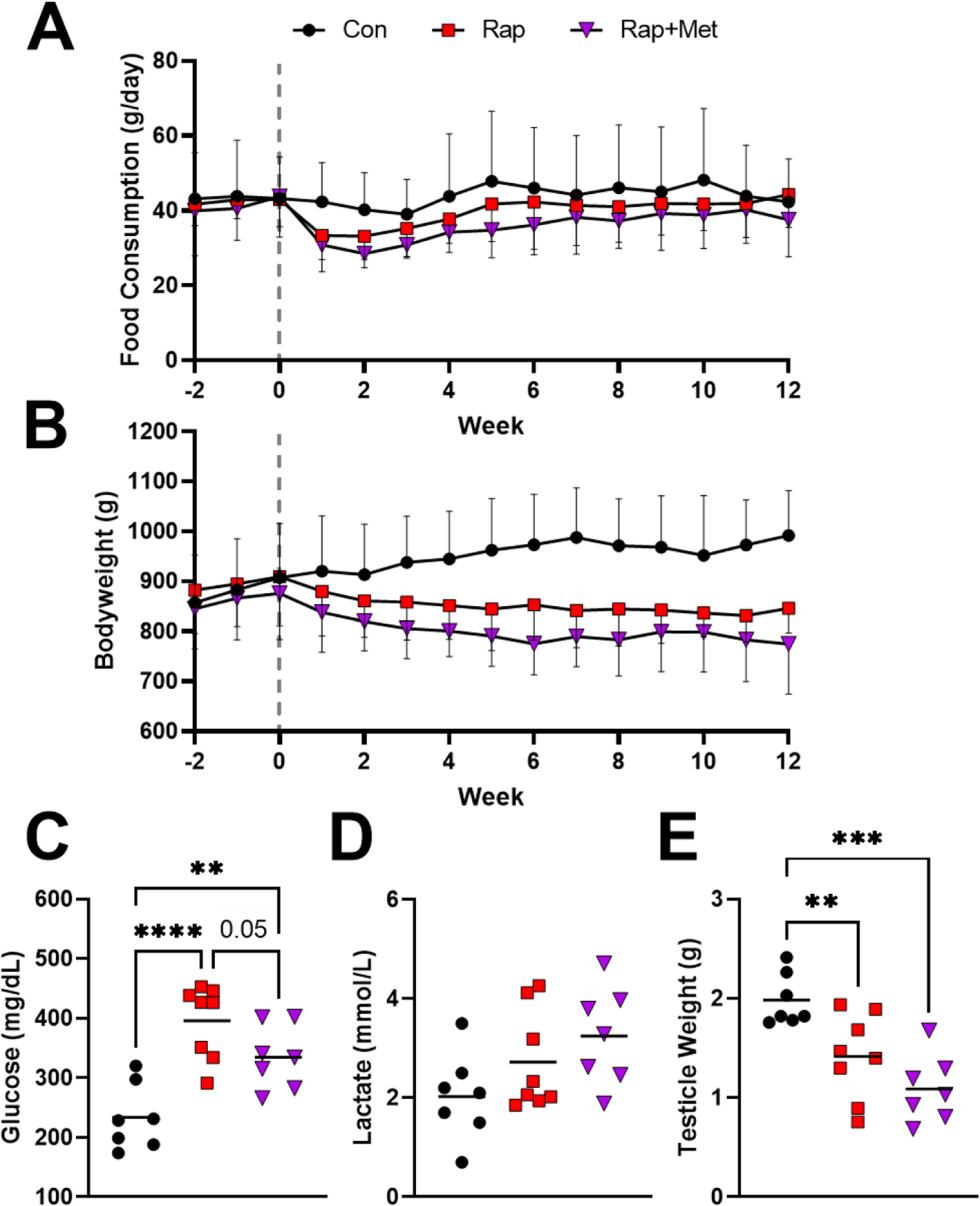
Characterization of animals on experimental diets. Food consumption (**A**) and bodyweight (**B**) of guinea pigs were recorded for the duration of the study (data presented as mean ± SD). Data analyzed using a two-way ANOVA to detect treatment by time interaction. Plasma glucose (**C**), lactate (**D**), and testicle weight (**E**) are shown. Data were analyzed using a one-way ANOVA to detect differences between groups for all variables besides lactate, for which differences were determined using the non-parametric Kruskal-Wallis test with Dunn’s multiple comparison test. **P < 0.01 vs Con, ***P < 0.001 vs Con, ****P < 0.0001 vs Con

Figure 1A shows the average daily food consumption per week of standard diet or standard diet enriched with Rap (14 ppm) or Rap+Met (14 + 1000 ppm). The average daily intakes of Rap and Met based on food consumption and dietary concentration are reported in Table 1. Compared to control, there was decreased food consumption in guinea pigs receiving Rap+Met (14 + 1000 ppm) during week 2 (*P* = 0.04). There were no significant differences between treatments. Despite largely matching food intake, there was a significant effect for treatment (*P* = 0.004) and an interaction between time and treatment (*P* < 0.0001) on bodyweight. Dietary Rap+Met (*P* = 0.01) and Rap-treated guinea pigs (*P* = 0.02) weighed less than control starting at week 3 and week 4, respectively, until the end of the study (Fig. 1B). At time of death, dietary Rap (*P* = 0.002) and Rap+Met-treated guinea pigs (*P* = 0.001) weighed 15% and 22% less than control.

**Table 1:** Average consumption of rapamycin and metformin. Using the concentration of rapamycin and metformin from the diet analysis, the average doses were calculated for each group in mg of drug per kg body weight per day (mg/kg/day). *N* = 7–8 per group. Data are presented as mean ± SD

	Experimental Diet	
	Rapamycin	Rapamycin+Metformin
Rapamycin consumed (mg/kg/day)	0.72 ± 0.09	0.68 ± 0.08
Metformin consumed (mg/kg/day)	-	45 ± 5.6

Compared to control, treatment with dietary Rap (14 ppm) and Rap+Met (14 + 1000 ppm) increased (*P* < 0.01) plasma glucose (Control, 234 ± 55 mg/dL, vs Rap, 396± 61 mg/dL, vs Rap+Met, 334 ± 53 mg/dL) while the addition of Met to Rap lowered plasma glucose compared to Rap alone (*P* = 0.05; Fig. 1C). Lactate concentration trended to be elevated by 66% in Rap+Met-treated guinea pigs, only (*P* = 0.1; Fig. 1D). Testicle weight in guinea pigs receiving Rap (*P* = 0.01) and Rap+Met (*P* = 0.0004) were 27% and 44% lower than control, respectively, suggesting gonadal atrophy (Fig. 1E). We analyzed blood for the circulating Rap and Met concentrations ~ 3 h after food had been removed from the cage (Table 2). This timing aligns with a measurement of peak circulating Rap and Met. We show that experimental diets were sufficient to increase Rap and Met concentrations in the blood, and that Rap values were not different when providing diets individually or in combination. In control animals, circulating levels of Rap or Met were below detectable limits.

**Table 2:** Concentrations of rapamycin and metformin in circulation. Whole blood was collected ~ 3 h after food had been removed from the cages of guinea pigs and was analyzed for rapamycin and metformin concentration by tandem HPLC/MS. *N* = 4 per group. The blood concentration of rapamycin and metformin in the control group were below the detectable limits of the HPLC/MS/MS. Data are presented as mean ± SD

	Experimental Diet		
	Control	Rapamycin	Rapamycin+Metformin
Circulating rapamycin (ng/mL)	< 0.5 ± 0	72 ± 8	78 ± 10
Circulating metformin (ng/mL)	< 4 ± 0	-	282 ± 54

### Dietary rapamycin (14 ppm) and rapamycin+metformin (14 + 1000 ppm) treatment exacerbated the age-related progression of OA

**Fig. 2:**
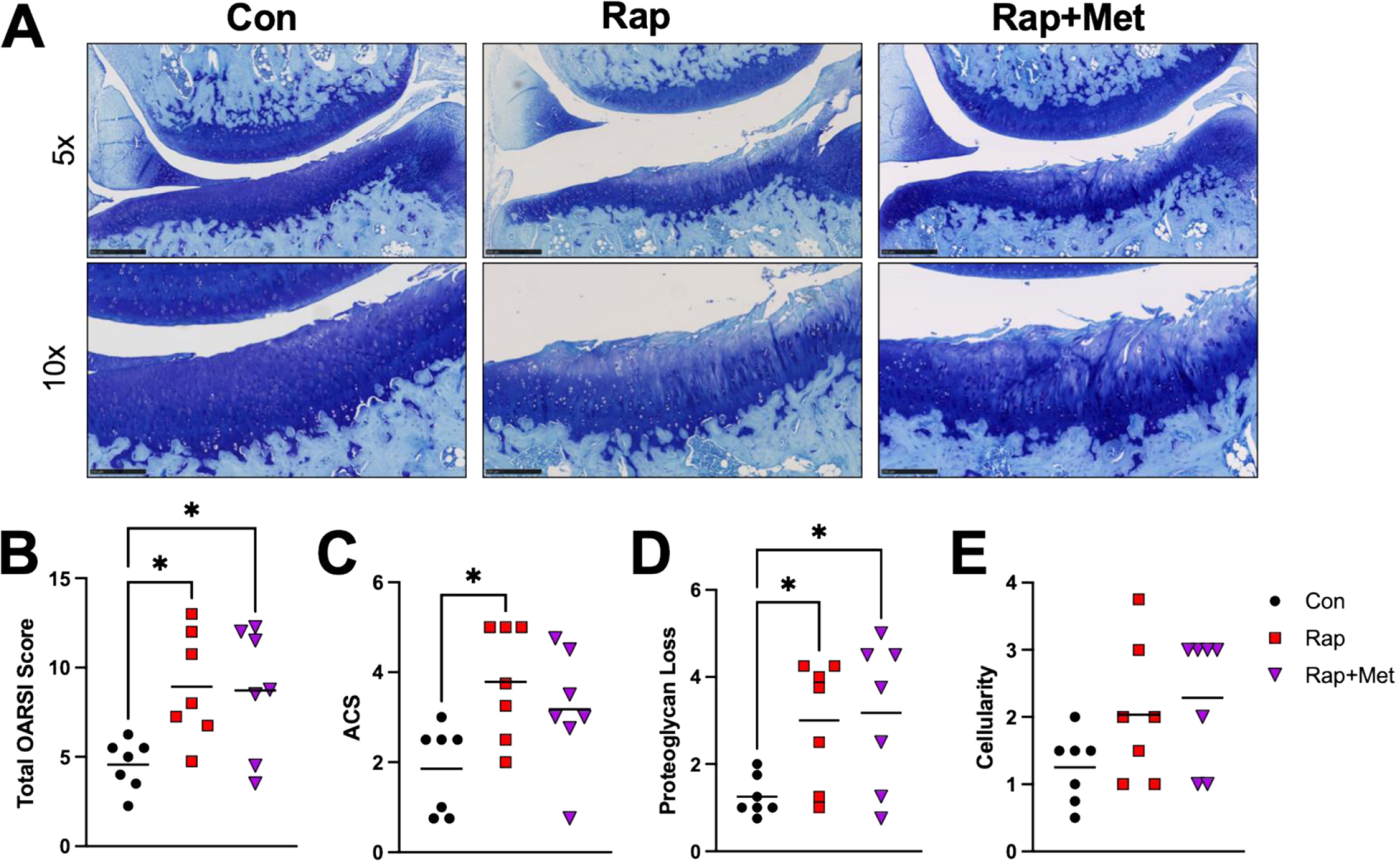
Rapamycin and rapamycin plus metformin worsened primary OA. Representative images of histology from the medial tibia are shown for each group (**A**; scale bars are 0.5 mm and 0.25 mm in 5× and 10× images, respectively). Histological images were graded for total OARSI score (**B**; *n* = 7 per group). The individual scores for articular cartilage structure (**C**), proteoglycan loss (**D**), and cellularity (**E**) are also shown. Data analyzed using a one-way ANOVA to detect differences between groups for all variables besides ACS and cellularity, for which differences were determined using the non-parametric Kruskal-Wallis test with Dunn’s multiple comparison test. *P < 0.05 vs Con

Consistent with the age-related progression of mild to moderate OA in guinea pigs, we observed an increase in medial tibial total OARSI score from 5 to 8 months (*P* = 0.03; Figure S1A-B). Surprisingly, dietary Rap and Rap+Met treatment resulted in a ~ 2-fold increase in total OARSI score in the medial tibial plateau compared to 8 month old, age-matched control (*P* = 0.02 for both Rap and Rap+Met; Fig. 2B). This was driven by increased scores for articular cartilage structure (*P* = 0.03 for Rap, *P* = 0.1 for Rap+Met; Fig. 2C) and proteoglycan loss (*P* = 0.02 for Rap and Rap+Met; Fig. 2D), while there was no differences between groups for cellularity (Fig. 2E). We observed minimal tidemark duplication and no osteophyte formation across groups. OA scores in lateral tibia or medial or lateral femur were lower than the medial tibial plateau and there was no significant effect of Rap or Rap+Met on the OARSI score for these joint compartments (Figure S1C).

### OA pathology was correlated to plasma glucose, bodyweight, and testicle weight

**Fig. 3:**
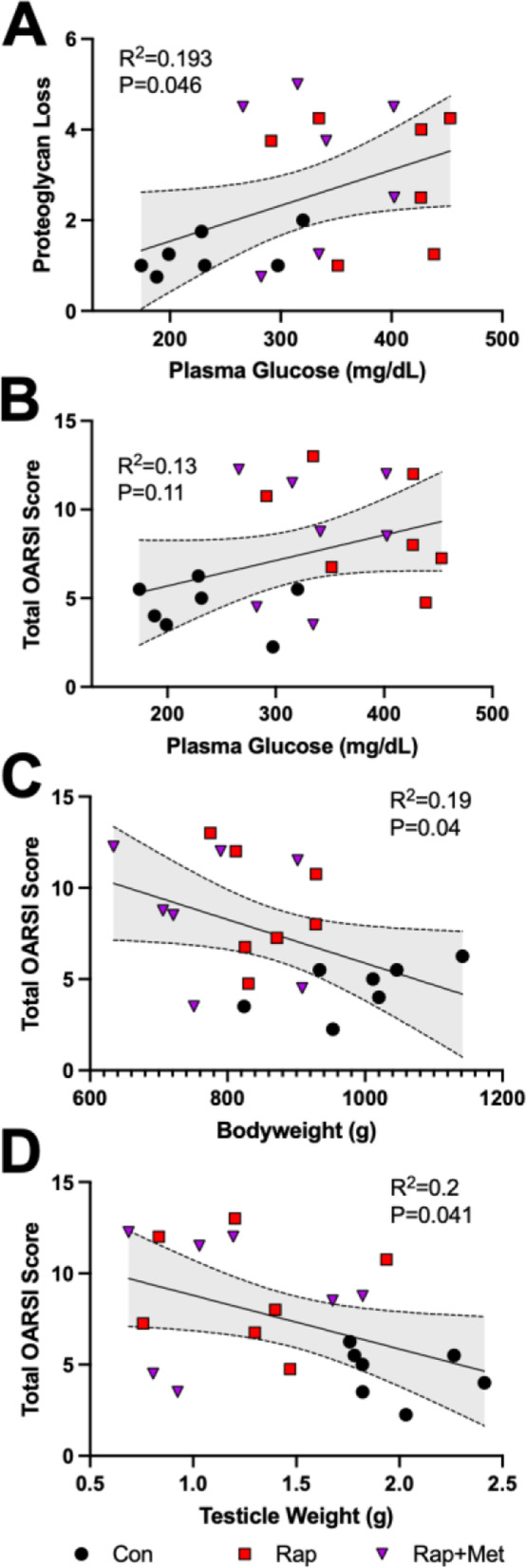
Proteoglycan loss correlated with hyperglycemia. Correlations between proteoglycan loss and plasma glucose (**A**), bodyweight and total OARSI score (**B**), and testicle weight and total OARSI score (**C**) are shown. Data analyzed using the Pearson correlation. Shaded bands represent 95% CI

Because dietary Rap- (14 ppm) and Rap+Met- (14 + 1000 ppm) treated guinea pigs displayed several common side effects of Rap, including increased plasma glucose, testicular atrophy, and decreased bodyweight, we evaluated the relationship between these variables and measures of OA severity across all guinea pigs. Plasma glucose was positively correlated to proteoglycan loss (*R*^2^ = 0.19; *P* = 0.04; Fig. 3A), and total OARSI score was negatively correlated with both bodyweight (*R*^2^ = 0.19; *P* = 0.04; Fig. 3B) and testicle weight (*R*^2^ = 0.20; *P* = 0.04; Fig. 3C). However, because testicle weight and bodyweight were also related (data not shown), the individual contribution of these variables cannot be distinguished.

### Effects of dietary rapamycin (14 ppm) and rapamycin+metformin (14 + 1000 ppm) on mTOR, AMPK, and protease expression

**Fig. 4:**
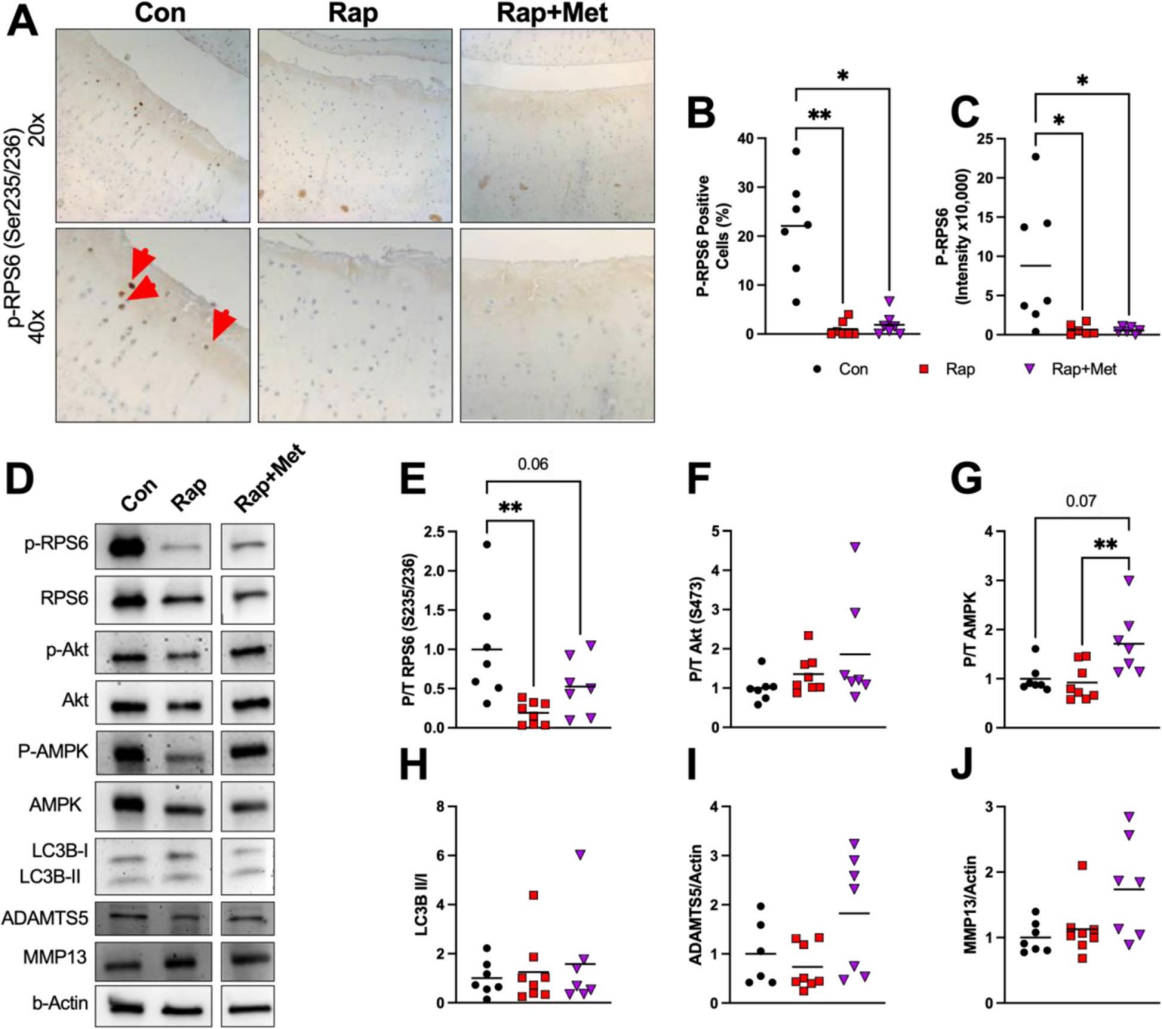
Rapamycin and rapamycin plus metformin inhibited mTORC1 but had no effect on mTORC2 or autophagy. IHC was performed on the medial tibia for P-RPS6 (**A**; *n* = 7 per group) and quantified as percent positive cells (**B**) and mean integrated intensity (**C**). Red arrowheads indicate cells staining positive for P-RPS6. Western blot was performed on glenohumeral cartilage (**D**) for P-RPS6 (**E**), P-Akt (**F**), P-AMPK (**G**), LC3B (**H**), ADAMTS5 (**I**), and MMP13 (**J**). Data analyzed using a one-way ANOVA to detect differences between groups for all variables except P-RPS6 (cells+), P-Akt, P-AMPK, LC3B, ADAMTS5, and MMP13, for which differences were determined by the non-parametric Kruskal-Wallis test with Dunn’s multiple comparison test. *n* = 8 per group for Rap and *n* = 7 per group for Con and Rap+Met. Images are outlined in black to show that, while each band is from the same blot, bands were selected for presentation to best represent the mean change. *P < 0.05 vs Con, **P < 0.01 vs Con

To evaluate mTORC1 signaling in articular cartilage, we measured the phosphorylation of ribosomal protein S6 (P-RPS6) at Ser235/236 using IHC and western blotting. Representative images of P-RPS6 IHC are shown in Fig. 4A. P-RPS6 was decreased by 90–95% in the medial tibial articular cartilage of Rap- and Rap+Met-treated guinea pigs as assessed by percentage of P-RPS6-positive cells (*P* = 0.001 for Rap, *P* = 0.01 for Rap+Met; Fig. 4B), and by staining intensity (*P* = 0.03 for both; Fig. 4C). mTORC1 inhibition was further supported by an 81% lower ratio of phosphorylated to total RPS6 in glenohumeral cartilage from Rap (*P* = 0.007; Fig. 4E) while Rap+Met trended to decrease RPS6 phosphorylation by 48% (*P* = 0.1). There was a non-signficant increase in the phosphorylation of the mTORC2 substrate Akt at Ser473 in Rap or Rap+Met compared to control (Fig. 4F; *P* = 0.1). AMPK activation was measured using western blot to assess phosphorylation of AMPK at Thr172 (P-AMPK). P-AMPK was not changed by Rap alone (*P* = 0.9; Fig. 4G) but tended to be elevated by 77% by Rap+Met (*P* = 0.07). Notably, AMPK phosphorylation was elevated in Rap+Met compared to Rap alone (*P* = 0.009). Rap or Rap+Met did not significantly change the conversion of LC3B I to II (*P* > 0.99 for both; Fig. 4H). A disintegrin and metalloproteinase with thrombospondin motifs 5 (ADAMTS5; Fig. 4I: *P* = 0.99 for Rap, *P* = 0.05 for Rap+Met) and matrix metalloproteinase 13 (MMP13; Fig. 4J: *P* = 0.99 for Rap, *P* = 0.1 for Rap+Met) was unchanged by Rap but trended higher in Rap+Met. Unless otherwise noted, there were no significant differences between Rap and Rap+Met.

### Dietary rapamycin (14 ppm) and rapamycin+metformin (14 + 1000 ppm) decreased subchondral and diaphyseal bone thickness

**Fig. 5:**
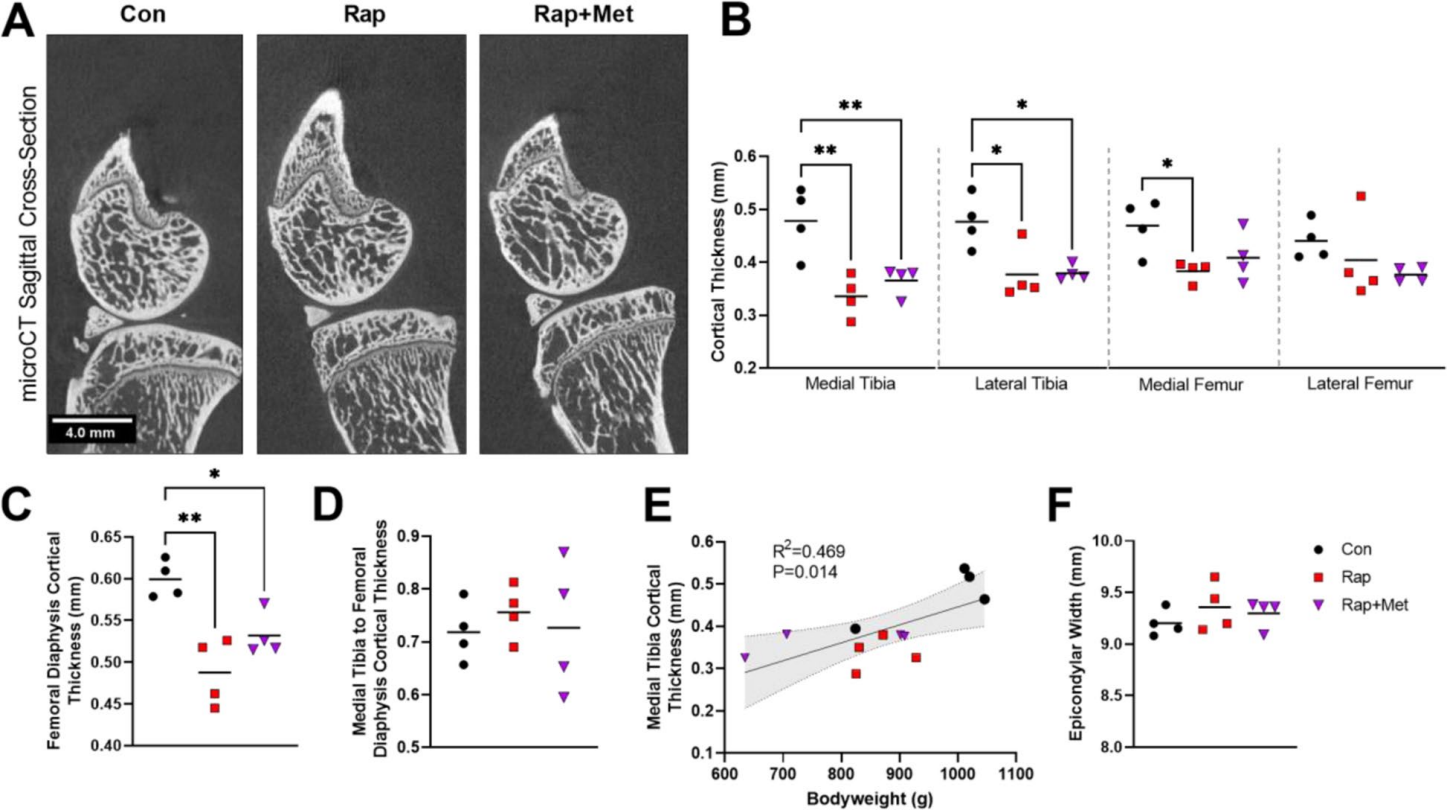
Decreased subchondral bone thickness in rapamycin and rapamycin plus metformin-treated guinea pigs. Representative microCT sagittal cross sections from the medial aspect of the joint are shown (**A**). Subchondral cortical thickness was measured in the medial and lateral tibial plateaus and femoral condyles (**B**), and cortical thickness was measured in the femoral diaphysis (**C**). Medial tibial cortical thickness relative to femoral diaphyseal cortical thickness was found to be similar between groups (**D**). Medial tibial cortical thickness was highly correlated to bodyweight (**E**). Femoral epicondylar width was found to be similar between groups (**F**). *N* = 4 per group. Data analyzed using a one-way ANOVA to detect differences between groups. Correlations assessed by Pearson correlation. Shaded bands represent 95% CI. *P < 0.05 vs Con, **P < 0.01 vs Con

Representative microCT images shown in Fig. 5A were used to quantify the effect of experimental diets on subchondral bone parameters. Mean subchondral cortical thickness was decreased by dietary Rap and Rap+Met in the medial (29%, *P* = 0.005 for Rap; 23%, *P* = 0.01 for Rap+Met) and lateral (21% for Rap; 20% for Rap+Met; *P* = 0.02 for both) tibia (Fig. 5B). Rap decreased cortical thickness in the medial femur (18%, *P* = 0.04). Rap and Rap+Met decreased trabecular spacing by 15% and 16%, respectively, in the lateral tibia only (*P* = 0.006 for both; Figure S2B). Trabecular thickness, trabecular spacing in other compartments, and bone volume fraction were not affected by any experimental diets (Figures S2A-C). Further investigation revealed that cortical thickness at the femoral diaphysis was also decreased by Rap (*P* = 0.001) and Rap+Met (*P* = 0.02; Fig. 5C), and this change was proportionate to the decrease observed in the medial tibial subchondral bone (Fig. 5D). Further, medial tibial cortical thickness was correlated to bodyweight (*R*^2^ = 0.47, *P* = 0.01; Fig. 5E), suggesting the smaller body mass of Rap- and Rap+Met-treated guinea pigs may have contributed to decreased cortical thickness. Femoral epicondylar width (Fig. 5F) was not statistically different between groups (Rap, *P* = 0.42; Rap+Met, *P* = 0.45), suggesting our treatments did not affect skeletal development.

## Discussion

The purpose of this study was to test if dietary Rap (14 ppm) or Rap+Met (14 + 1000 ppm) could delay the onset of age-related OA in the outbred Dunkin-Hartley guinea pig. We found that at concentrations shown to extend lifespan, dietary Rap (14 ppm) and Rap+Met (14 + 1000 ppm) inhibited mTORC1 but not mTORC2 signaling in articular cartilage, and Rap+Met increased AMPK phosphorylation. Surprisingly, guinea pigs treated with dietary Rap (14 ppm), with or without Met (± 1000 ppm), developed greater age-related OA compared to control. Guinea pigs receiving dietary Rap (14 ppm) and Rap+Met (14 + 1000 ppm) also displayed increased plasma glucose, which correlated with proteoglycan loss. These findings indicate that off-target hyperglycemic side effects of dietary Rap (14 ppm) are associated with greater hallmarks of OA pathology. Further, in the face of these Rap-induced side effects, mTORC1 inhibition may not slow the progression of age-related OA in Dunkin Hartley guinea pigs.

Despite inhibiting mTORC1 in articular cartilage, our findings indicate that guinea pigs treated with dietary Rap, with or without Met, had exacerbated age-related OA in the medial tibial plateau. Further, dietary Rap- (14 ppm) and Rap+Met- (14 + 1000 ppm) treated guinea pigs had greater total OARSI scores than age-matched control animals even though they weighed less, which is contrary to previous work where lower body weight was accompanied by lower OA scores in guinea pigs [30]. Although there is precedent that mTORC1 inhibition by intra-articular injection of Rap is associated with exacerbated temporomandibular joint (TMJ) OA [50], our findings were opposite of our original hypothesis and previous results using Rap in injury-induced models of knee OA [19, 20]. In our study, Rap and Rap+Met treatment inhibited mTORC1 but not mTORC2 in articular cartilage. Previous work has shown that deleting articular cartilage mTOR [22] or treating with Rap [19, 20] or Torin-1 [21] can attenuate injury or chemical-induced OA in mice and rabbits. These non-selective genetic and pharmacological methods likely disrupt the entire mTOR kinase and therefore could inhibit both mTORC1 and mTORC2 signaling. However, this remains speculative as mTORC2 signaling was not evaluated in these previous studies, and it continues to be unknown if mTORC2 inhibition is necessary for protection against either primary or secondary OA. Further investigation is needed to resolve the role of each mTOR complex in the initiation, progression, and treatment of both primary and secondary OA.

While the dose of Rap in guinea pigs was similar to the dose shown to protect against injury-induced OA in mice (0.7 vs 1 mg of rapamycin per kg body weight per day in guinea pigs vs. mice) [19], after correcting for differences in pharmacokinetics between animals with vastly different body weight, allometric scaling [51] suggests the guinea pig equivalent dose was double that of mice. However, the guinea pigs in the current study received the same dietary concentration (14 ppm) and achieved similar circulating Rap concentrations shown to extend lifespan and decrease select age-related pathologies in mice involved in the NIA Interventions Testing Program [14]. Further, allometric scaling indicates that dietary Rap (14 ppm) elicited similar doses of rapamycin between guinea pigs in the current study and long-lived mice in the ITP.

Despite its lifespan and potential healthspan-extending effects, chronic Rap treatment is commonly associated with several metabolic and immunological side effects across diverse species including glucose intolerance, new onset diabetes, hypertriglyceridemia, immunosuppression, testicular atrophy, lower body weight, and cataracts [18, 45, 52]. Consistent with this, we showed that 12 weeks of dietary Rap (14 ppm) and Rap+Met (14 + 1000 ppm) was accompanied by increased plasma glucose, testicular atrophy, and lower body weight compared to controls. The glucose values induced by dietary Rap (14 ppm) and Rap+Met (14 + 1000 ppm) are comparable to previous studies that have induced diabetes in guinea pigs [53, 54]. While there are no criteria to confirm a diabetes diagnosis in guinea pigs and we cannot directly compare between different studies, these findings are in line with rapamycin promoting new onset diabetes.

Despite increasing AMPK activity in articular cartilage and partially restoring glucose levels compared to Rap alone, the addition of the antihyperglycemic drug Met to Rap did not offer significant protection against the detrimental effects of dietary Rap on OA pathology. In our study, medial tibial proteoglycan loss was correlated with plasma glucose, and we propose that Rap-induced hyperglycemia may have contributed to worsened OA following dietary Rap. In support of this hypothesis, diabetic mice show more severe OA after injury and high glucose suppresses autophagy activation and can increase MMP13, IL-6, and NFkB in chondrocytes [55–61]. Rap has been implicated in attenuating secondary OA by increasing autophagy and decreasing protease expression [19, 20]. In our study, we observed no effect by any treatment on the lipidation of the autophagy marker LC3B while Rap+Met trended to increase ADAMTS5 and MMP13 in glenohumeral cartilage. Therefore, the inability to increase markers of autophagy and decrease proteases may be contributing factors to why our treatments did not protect and even worsened OA during aging and hyperglycemia.

Intermittent intraperitoneal injections of Rap lowered glucose and injury induced OA severity in diabetic mice [62]. It is possible in the current study that Rap did offer partial protection against hyperglycemic stress but still resulted in greater OA pathology than control. However, this remains speculative as we did not have a group exposed to hyperglycemic stress alone. Previous work suggests Rap can have divergent effects where it is beneficial in some diabetic models but causes adverse side effects in metabolically healthy models [18, 63]. Collectively, these data suggest that the adverse metabolic side-effects of dietary Rap treatment are associated with a deleterious impact on primary OA pathology and could limit the utility of chronic Rap as a healthspan extending treatment.

Treatment with dietary Rap and Rap+Met also decreased subchondral cortical bone thickness in the medial and lateral tibia and the femoral diaphysis. As bone growth in guinea pigs ceases by 4 months [64], and epicondylar width was not different between groups, the differences in bone thickness were likely not the result of disrupted development. Decreased subchondral thickness was only observed in the tibia. Intra-articular injection of Rap into the TMJ caused subchondral bone loss by inhibiting pre-osteoblast proliferation [50], and Rap treatment also decreased osteoblast differentiation and bone matrix synthesis [65], which supports the idea that Rap can act directly on the bone to decrease thickness. However, we also found that subchondral thickness was highly correlated to bodyweight. This is in line with Wolff’s law and agrees with previous findings where bodyweight restriction decreased cortical bone thickness in the femoral diaphysis [66]. Therefore, both local and systemic effects of Rap likely contributed to reduced cortical bone thickness.

Although we provide new insight into the role of mTOR during primary OA progression, we recognize some study limitations. While the guinea pig is an excellent model of primary OA, molecular probes are seldom designed for reactivity with guinea pigs. Due to reactivity issues with IHC in guinea pig cartilage (Figure S3), some of our analyses relied on western blot from glenohumeral cartilage. Although guinea pigs also develop mild glenohumeral OA [31], this is not the site at which we measured OA pathology. Our study could not conclusively determine if the deleterious effects of dietary Rap (14 ppm) stemmed from its direct effects on the joint or off-target effects on other tissues. However, our data suggest hyperglycemia induced by off-target actions of Rap was associated with greater proteoglycan loss. This relationship could have been further strengthened by the addition of a group of guinea pigs exposed to hyperglycemic stress without Rap. The Dunkin Hartley guinea pig is an outbred model of primary OA which leads to inherent variability. While this could be perceived as a limitation, we contend that the variability and the choice of animal model adds translational value since this more closely recapitulates the genetic diversity and OA heterogeneity in humans. Despite these limitations, this does not detract from the findings that guinea pigs treated with dietary Rap (14 ppm) and Rap+Met (14 + 1000 ppm) had worse OA. Further, the presence of largely overlapping and consistent deleterious outcomes in both groups receiving dietary Rap increases our confidence that the side effects accompanying Rap contribute to worsened primary OA.

## Conclusion

In summary, we have shown that at doses previously shown to extend lifespan, dietary Rap (14 ppm) and Rap+Met (14 + 1000 ppm) caused hyperglycemia and was associated with aggravated OA in Dunkin Hartley guinea pigs despite inhibiting mTORC1 in articular cartilage. Treatments that extend lifespan without a proportionate delay in age-related chronic diseases and disabilities are counter to the concept of healthspan extension. Our findings that guinea pigs treated with dietary Rap had worse OA pathology raises concerns regarding the efficacy of dietary Rap as a life- and healthspan-extending agent. Additional work is needed to investigate the role of alternative routes of administration or Rap analogs that may capture the positive benefits of Rap while minimizing off-target effects. Our data also reveal that the contribution of mTOR in articular cartilage and chondrocyte metabolism is incompletely understood and additional research is needed to clarify the individual and combined role of mTORC1 and mTORC2 signaling in OA.

## Supplementary Information

**Additional file 1: Figure S1.** OA pathology increased from 5- to 8-months of age. A) Representative histological images of the medial tibial plateu of the knee joint from 5- and 8-month-old guinea pigs (scale bars are 0.5mm and 0.25mm in 5x and 10x images, respectively). B) Images were graded for total OARSI score and individual OARSI criteria. Comparisons for ACS were made using the non-parametric Mann-Whitney test. C) the lateral tibial plateau, medial femoral condyle and lateral femoral condyle were also graded for total OARSI score. Data *N* = 3 for 5-month and *N* = 7 for 8-month. *P < 0.05 vs Con. **Figure S2.** Trabecular bone changes in response to experimental diets. Trabecular thickness (A), spacing (B), and bone volume fraction (C) were measured using microCT. *N* = 4 per group. *P < 0.05 vs Con. **Figure S3.** Antibody reactivity with guinea pig articular cartilage was limited. Immunohistochemical staining was performed, and no reactivity was observed using primary antibodies against P-Akt Ser473 or P-AMPK Thr172.

## Data Availability

Data from this study are available from the corresponding author, Adam Konopka, upon reasonable request.

## Abbreviations

OA: Osteoarthritis
mTOR: Mechanistic target of rapamycin
AMPK: AMP-activated protein kinase
Rap: Rapamycin
Met: Metformin
mTORC1: mTOR complex I
mTORC2: mTOR complex II
NBF: Neutral buffered formalin
μCT: Micro-computed tomography
ROI: Region of interest
IHC: Immunohistochemistry
OARSI: Osteoarthritis research society international
SD: Standard deviation
RPS6: Ribosomal protein S6
ADAMTS5: A disintegrin and metalloproteinase with thrombospondin motifs 5
MMP13: Matrix metalloproteinase 13
TMJ: Temporomandibular joint
IL-6: Interleukin 6
NFkB: Nuclear factor kappa-light-chain-enhancer of activated B-cells
ECM: Extracellular matrix
