# Correction to: Rapamycin-induced hyperglycemia is associated with exacerbated age-related osteoarthritis

**DOI:** 10.1186/s13075-022-02718-9

**Published:** 2022-01-11

**Authors:** Dennis M. Minton, Christian J. Elliehausen, Martin A. Javors, Kelly S. Santangelo, Adam R. Konopka

**Affiliations:** 1 Division of Geriatrics and Gerontology, Department of Medicine, University of Wisconsin-Madison, Madison, WI USA; 2 Department of Kinesiology, University of Illinois at Urbana-Champaign, Champaign, IL USA; 3 Departments of Psychiatry and Pharmacology, University of Texas Health Science Center at San Antonio, San Antonio, TX USA; 4 Department of Microbiology, Immunology, Pathology, Colorado State University, Fort Collins, CO USA; 5 Geriatric Research, Education, and Clinical Center, William S. Middleton Memorial Veterans Hospital, Madison, WI USA

**Correction to: Arthritis Res Ther 23, 253 (2021)**

**https://doi.org/10.1186/s13075-021-02637-1**

Following publication of the original article [1], the authors reported a spelling error in the fourth author’s last name. Author Kelly S. Santangelo has been corrected.

The original article [1] has been updated.

## References

[1] Minton DM, Elliehausen CJ, Javors MA (2021). Rapamycin-induced hyperglycemia is associated with exacerbated age-related osteoarthritis. Arthritis Res Ther.

