# Supplementary material for: Rapamycin-induced hyperglycemia is associated with exacerbated age-related osteoarthritis
Source: Arthritis Res Ther. 2021 Oct 7;23:253. doi: 10.1186/s13075-021-02637-1 (PMC8495984; doi:10.1186/s13075-021-02637-1)

**Supplementary Material**

Supplementary Figure Legends

**Figure S1: OA pathology increased from 5- to 8-months of age.** A) Representative histological images of the medial tibial plateu of the knee joint from 5- and 8-month-old guinea pigs (scale bars are 0.5mm and 0.25mm in 5x and 10x images, respectively). B) Images were graded for total OARSI score and individual OARSI criteria. Comparisons for ACS were made using the non-parametric Mann-Whitney test. C) the lateral tibial plateau, medial femoral condyle and lateral femoral condyle were also graded for total OARSI score. Data N=3 for 5-month and N=7 for 8-month. *P<0.05 vs Con.

**Figure S2: Trabecular bone changes in response to experimental diets.** Trabecular thickness (A), spacing (B), and bone volume fraction (C) were measured using microCT. N=4 per group. *P<0.05 vs Con.

**Figure S3: Antibody reactivity with guinea pig articular cartilage was limited.** Immunohistochemical staining was performed, and no reactivity was observed using primary antibodies against P-Akt Ser473 or P-AMPK Thr172.

Supplementary Figures

**Figure S1**


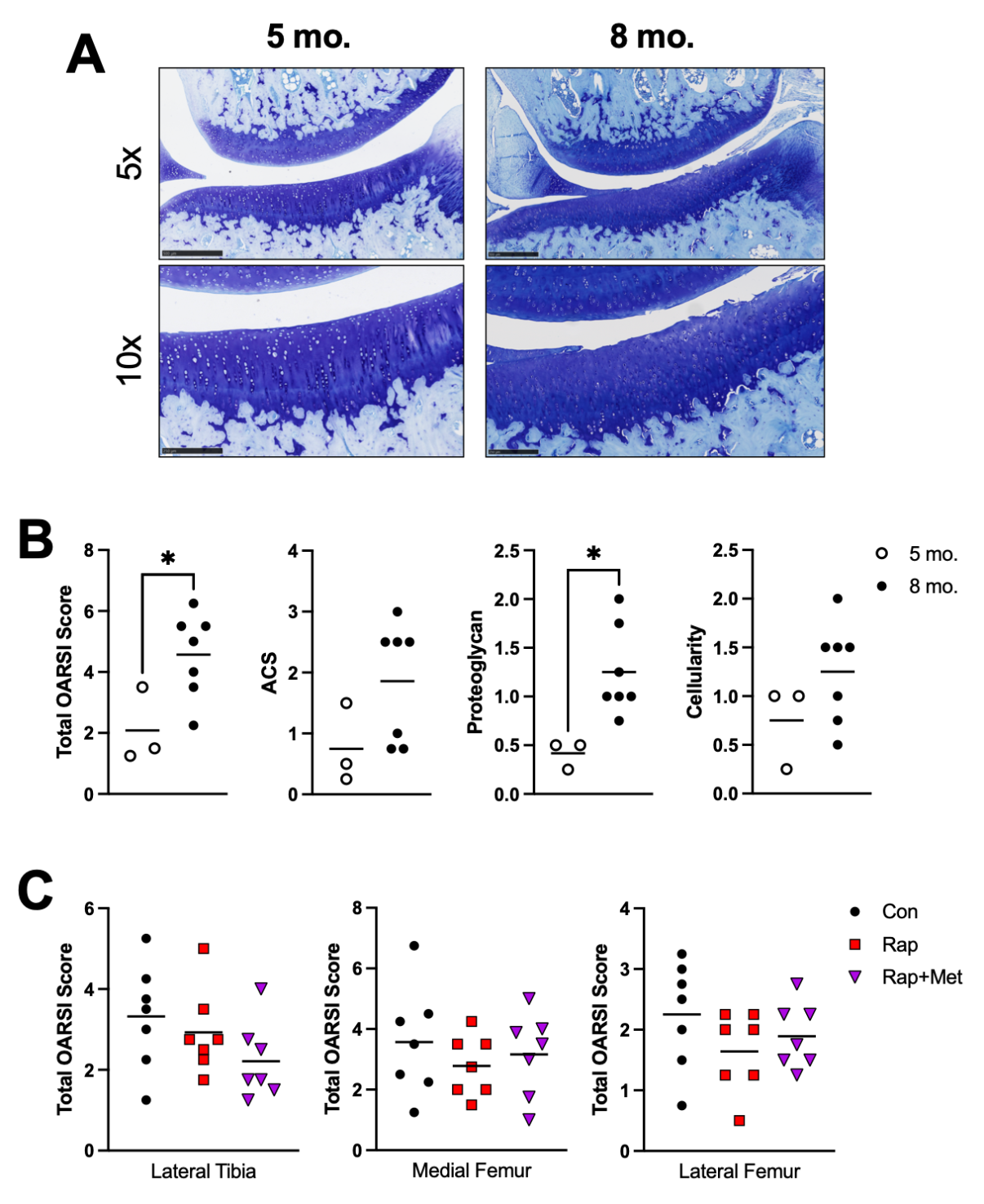


**Figure S2**


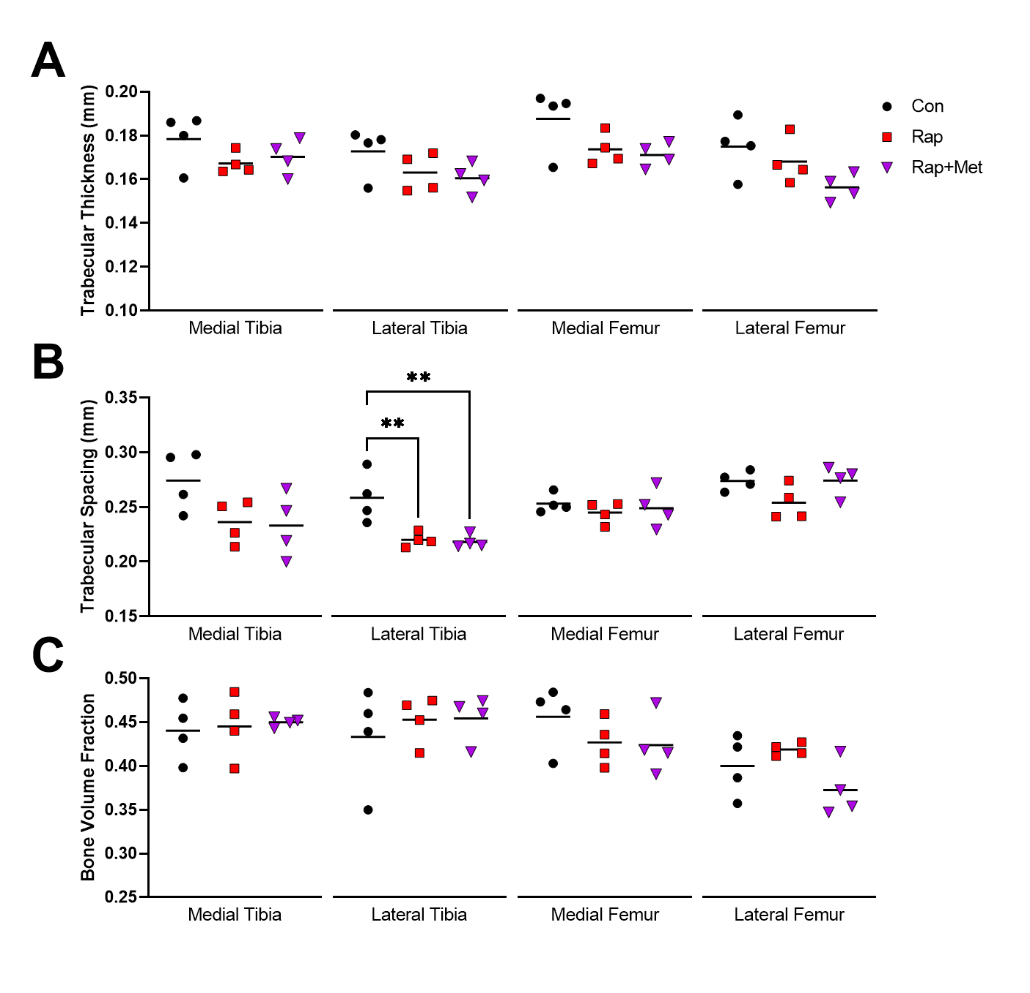


**Figure S3**


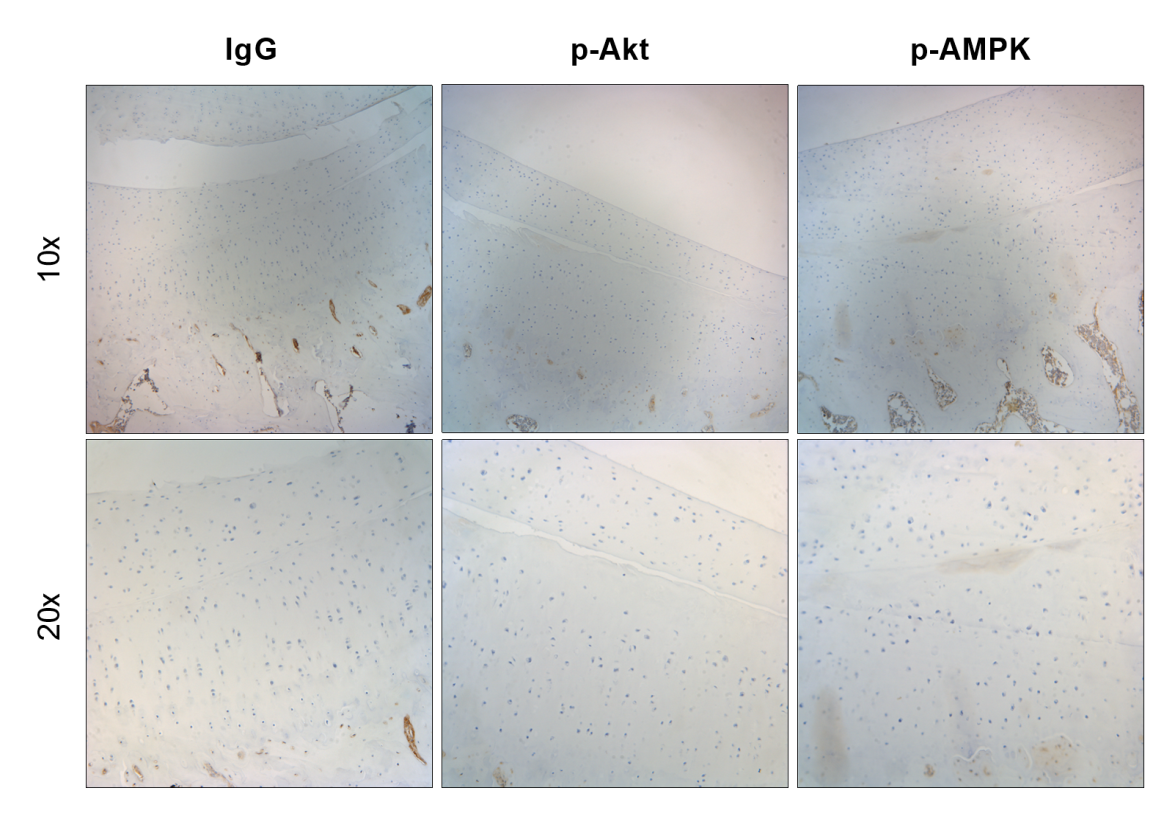

Supplement: Supplementary file 1 — Additional file 1: Figure S1. OA pathology increased from 5- to 8-months of age. A) Representative histological images of the medial tibial plateu of the knee joint from 5- and 8-month-old guinea pigs (scale bars are 0.5mm and 0.25mm in 5x and 10x images, respectively). B) Images were graded for total OARSI score and individual OARSI criteria. Comparisons for ACS were made using the non-parametric Mann-Whitney test. C) the lateral tibial plateau, medial femoral condyle and lateral femoral condyle were also graded for total OARSI score. Data N = 3 for 5-month and N = 7 for 8-month. *P < 0.05 vs Con. Figure S2. Trabecular bone changes in response to experimental diets. Trabecular thickness (A), spacing (B), and bone volume fraction (C) were measured using microCT. N = 4 per group. *P < 0.05 vs Con. Figure S3. Antibody reactivity with guinea pig articular cartilage was limited. Immunohistochemical staining was performed, and no reactivity was observed using primary antibodies against P-Akt Ser473 or P-AMPK Thr172. [file 13075_2021_2637_MOESM1_ESM.docx]
